# Expression of Class II Major Histocompatibility Complex Antigens on Adult T Cells in *Xenopus* is Metamorphosis- Dependent

**DOI:** 10.1155/1990/25197

**Published:** 1990

**Authors:** Louise A. Rollins-Smith, Patrick Blair

**Affiliations:** Department of Pediatrics Division of Pediatric Immunology and Rheumatology Vanderbilt University School of Medicine Nashville Tennessee 37232 USA

## Abstract

Class II major histocompatibility complex (MHC) antigens are expressed predominantly on B lymphocytes and macrophages of tadpoles of the South African clawed frog, *Xenopus laevis*, as is the pattern in lymphocyte populations of most mammals. However, unlike most mammals, young postmetamorphic frogs show expression of class II MHC antigens on a high proportion of thymocytes and most peripheral T and B lymphocytes. Using the J-strain of *Xenopus* and the anticlass II monoclonal antibody, 14A2, we have studied, by indirect immunofluorescence, whether inhibition of metamorphosis would alter the pattern of expression of class II antigens during ontogeny. In control animals, class II antigens were virtually absent from thymic lymphocytes and peripheral T cells of normal untreated larvae, but could be found in increasing numbers in both populations after metamorphosis (10-12 weeks of age). In contrast, larvae, whose metamorphosis was inhibited by treatment with sodium perchlorate, had relatively few class II^+^ thymic lymphocytes throughout the 6-month period of study, and the proportion of class II^+^ splenic lymphocytes was approximately equal to that of IgM^+^ B lymphocytes. Thus, perchlorate-treated animals retained the larval pattern of class II epression, suggesting that emergence of class II^+^ T cells is dependent on metamorphosis.


*Developmental Immunology*, 1990, Vol. 1, pp. 97–104
Reprints available directly from the publisher
Photocopying permitted by license only

© 1990 Harwood Academic Publishers GmbH
Printed in the United Kingdom

### Expression of Class II Major Histocompatibility Complex Antigens on Adult T Cells in *Xenopus* is Metamorphosis-Dependent

LOUISE A. ROLLINS-SMITH* and PATRICK BLAIR

*Department of Pediatrics, Division of Pediatric Immunology and Rheumatology, Vanderbilt University School of Medicine, Nashville, Tennessee 37232*

Class II major histocompatibility complex (MHC) antigens are expressed predominantly on B lymphocytes and macrophages of tadpoles of the South African clawed frog, *Xenopus laevis*, as is the pattern in lymphocyte populations of most mammals. However, unlike most mammals, young postmetamorphic frogs show expression of class II MHC antigens on a high proportion of thymocytes and most peripheral T and B lymphocytes. Using the J-strain of *Xenopus* and the anticlass II monoclonal antibody, 14A2, we have studied, by indirect immunofluorescence, whether inhibition of metamorphosis would alter the pattern of expression of class II antigens during ontogeny. In control animals, class II antigens were virtually absent from thymic lymphocytes and peripheral T cells of normal untreated larvae, but could be found in increasing numbers in both populations after metamorphosis (10–12 weeks of age). In contrast, larvae, whose metamorphosis was inhibited by treatment with sodium perchlorate, had relatively few class $II^+$ thymic lymphocytes throughout the 6-month period of study, and the proportion of class $II^+$ splenic lymphocytes was approximately equal to that of $IgM^+$ B lymphocytes. Thus, perchlorate-treated animals retained the larval pattern of class II expression, suggesting that emergence of class $II^+$ T cells is dependent on metamorphosis.

KEYWORDS: Class II MHC antigens, T lymphocytes, metamorphosis.

### INTRODUCTION

*Xenopus laevis* is the most primitive vertebrate with a defined MHC (reviewed by Du Pasquier et al., 1989). As an amphibian, it is also unique among vertebrates because it undergoes metamorphosis. With this reorganization of nearly every major physiological system comes the activation of new gene programs and the appearance of new adult-specific molecules (Just et al., 1977, 1980; May and Knowland, 1980). As a result of metamorphosis, the immune system also undergoes a number of changes (reviewed in Flajnik et al., 1987, and Du Pasquier et al., 1989). Among them are changes in the pattern of expression of MHC molecules. Reagents that immunoprecipitate class I MHC molecules from adult cells reveal no classical class I molecules on tadpole cells (Flajnik et al., 1986). The class II molecules expressed by tadpoles and adults are the same (Flajnik et al., 1986), but they are expressed on different subpopulations of lymphocytes. In the tadpole, class II antigens are expressed on B lymphocytes and macrophages, whereas in the adult, they are expressed on some thymic lymphocytes and virtually all peripheral B and T lymphocytes (Du Pasquier and Flajnik, 1990; Flajnik et al., 1990). We have hypothesized that one way by which tadpoles could accommodate new adult-specific antigens would be to largely discard the larval immune system and develop a new one after metamorphosis. That is, metamorphosis would be characterized by a major loss of larval-type lymphocytes with new lymphopoiesis and expansion of the adult-type population after metamorphosis. This is a reasonable hypothesis in view of the well-studied changes in erythrocyte populations that occur at metamorphosis in anuran amphibians. Larval and adult erythrocytes differ in morphology and express different hemo-

*Corresponding author.

globins (reviewed by Broyles, 1981). In both *Rana catesbeiana* and *Xenopus laevis,* adult hemoglobin appears to be expressed at metamorphosis only in newly differentiating erythrocytes. Larval erythrocytes become biosynthetically inactive and soon disappear from circulation (Just et al., 1980; Dorn and Broyles, 1982; Flajnik and Du Pasquier, 1988). Here we present evidence that the adult-type class II$^+$ population of T lymphocytes that normally arises in young postmetamorphic frogs does not appear in metamorphosis-inhibited permanent larvae. This suggests that development of this population is metamorphosis-dependent.

### RESULTS

### A Comparison of Fluorescence-Positive Populations as Determined by Fluorescence Microscopy or Flow Cytometry

In several initial experiments, we determined the percentages of class II$^+$ and IgM$^+$ lymphocytes by both flow cytometry and conventional fluorescence microscopy. We showed (Table 1) that the percentages observed by both methods were nearly identical. This demonstrates that we are able to detect the same fluorescence-positive populations by fluorescence microscopy as detected by the potentially more sensitive flow cytometric method.

### Expression of Class II Antigens on Thymic and Splenic Lymphocytes During Ontogeny

Class II antigens were expressed on about 3–4% of thymocytes at larval stages 53–57 and metamorphic stages 62–66. By 3–4 months of age (approximately 1–2 months postmetamorphosis), the number of thymocytes expressing class II determinants had increased to about 19%. At 5–6 months of age, 32% of thymocytes expressed class II antigens. In contrast, the proportion of thymocytes staining positive for surface IgM remained low throughout ontogeny (Fig. 1). Thus, most class II$^+$ cells in the thymus are not B lymphocytes. Some macrophages in the thymus (by morphological criteria) are also class II$^+$, but they represent only 1–3% of the larval or adult thymocyte population by esterase staining (data not shown). Thus, the vast majority of class II$^+$ cells in the thymus are maturing T cells.

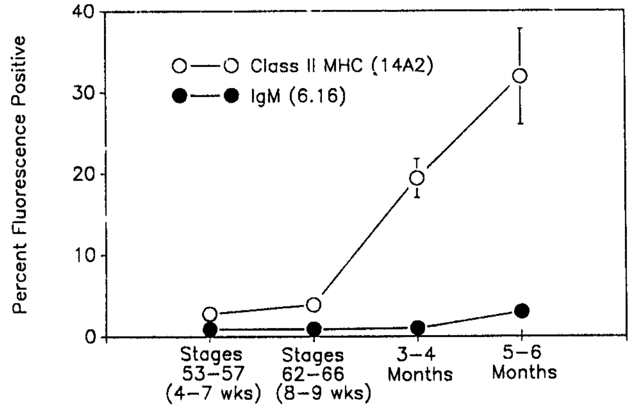

FIGURE 1. Ontogeny of expression of class II MHC antigens on thymocytes of J-strain frogs. Thymocytes were stained for class II antigens (O——O) using the monoclonal antibody designated 14A2 and surface IgM (●——●) using the monoclonal antibody designated 6.16. Both antibodies were used at a concentration of approximately 100–200 μg/ml and were followed by staining with a fluorescein-conjugated polyvalent goat antimouse Ig used at 100 μg/ml. Each data point represents the mean ± SE of 5–7 individuals or pools of animals. Metamorphosis occurred in these frogs at about 10–12 weeks of age.

TABLE 1
A Comparison of Fluorescence Positive Thymus and Spleen-Cell Populations as Determined by Fluorescence Microscopy or Flow Cytometry[a]

| Exp. No. | Treatment | Population | Percent Class II$^+$ by: Fluorescence microscopy | Percent Class II$^+$ by: Flow cytometry | Percent IgM$^+$ by: Fluorescence microscopy | Percent IgM$^+$ by: Flow cytometry |
|---|---|---|---|---|---|---|
| 1 | Untreated | Spleen | 45.2 | 44.9 | 34.4 | 35.0 |
| | | Thymus | 11.7 | 16.7 | 0.8 | 1.0 |
| | Perchlorate | Spleen | 33.3 | 32.7 | 25.0 | 25.2 |
| | | Thymus | 18.6 | 14.6 | ND | ND |
| 2 | Untreated | Spleen | 36.5 | 47.3 | 44.3 | 42.8 |
| | | Thymus | 17.0 | 19.0 | 1.5 | 1.5 |
| | Perchlorate | Spleen | 31.8 | 30.1 | 23.0 | 25.0 |
| | | Thymus | 11.5 | 10.7 | 2.0 | 2.8 |

[a]Indirect staining was done as described in Materials and Methods. For flow cytometry, cells were fixed after staining for at least 1 h in amphibian PBS containing 0.2% azide, 2% fetal calf serum, and 1% formalin. Approximately 5000 cells per sample were analyzed with an EPICS 753 flow cytometer (Coulter Electronics, Hialeah, FL). ND = not determined.

In the spleen, between 35–40% of lymphocytes expressed class II antigens during larval stages 53–57 and metamorphic stages 62–66. These percentages were virtually identical to the percentage of cells expressing surface IgM. This confirms the observation of Du Pasquier and Flajnik (1990) that class II antigens are expressed on B lymphocytes during larval life. At 5–6 months of age (2–3 months postmetamorphosis), the percent of class II$^+$ splenocytes had increased to about 72% while IgM$^+$ cells remained at about 42%. This suggests that class II$^+$ T lymphocytes had become a significant part of the splenic lymphocyte population (Fig. 2), as shown previously by Flajnik et al. (1990).

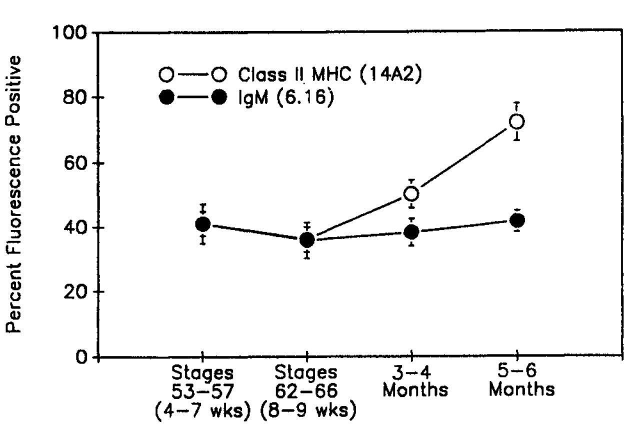

FIGURE 2. Ontogeny of expression of class II MHC antigens on lymphocytes in the spleen of J-strain frogs. Splenocytes were stained for class II antigens (O———O) and surface IgM (●———●), as described in the legend for Fig. 1. Each data point represents the mean ± SE of 5–7 individuals or pools of animals.

In contrast to the pattern shown in Figs. 1 and 2 for normal animals, perchlorate-treated frogs showed a much different pattern of expression of class II antigens during ontogeny. Thymocytes from perchlorate-treated frogs at 5–9 weeks of age, when normal control frogs are at larval and metamorphic stages, contained about the same number of class II$^+$ lymphocytes as the normal J-strain controls of the same age. By 3–4 months of age, the percent of class II$^+$ thymocytes had increased to about 11%, but did not increase further at 5–6 months of age, when normal controls contained about 32% class II$^+$ thymocytes (Fig. 3). In the spleen of perchlorate-treated frogs, class II$^+$ lymphocytes comprised about 25–40% of the total throughout the 6-month period of study. That proportion was virtually identical to the proportion of IgM$^+$ cells, suggesting that class II$^+$ cells in perchlorate-treated spleens are mostly B cells (Fig. 4). To confirm that class II$^+$ cells in perchlorate-treated spleens were B cells, we did several dual fluorescence experiments in which we stained directly for class II antigens and IgM on the same cells. In perchlorate-treated spleens, virtually all cells staining for class II were also IgM$^+$, whereas in normal controls of the same age, about 15% of the cells (i.e., class II$^+$ T cells) were class II$^+$ IgM$^-$ (Table 2).

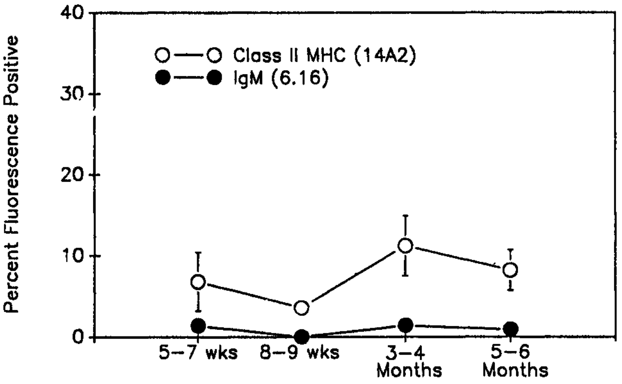

FIGURE 3. Ontogeny of expression of class II MHC antigens (O———O) and surface IgM (●———●) on thymocytes of perchlorate-blocked J-strain larvae. Staining was done as described in the legend of Fig. 1. Each data point represents the mean ± SE of 2–5 individuals or pools of animals (4–8 animals total).

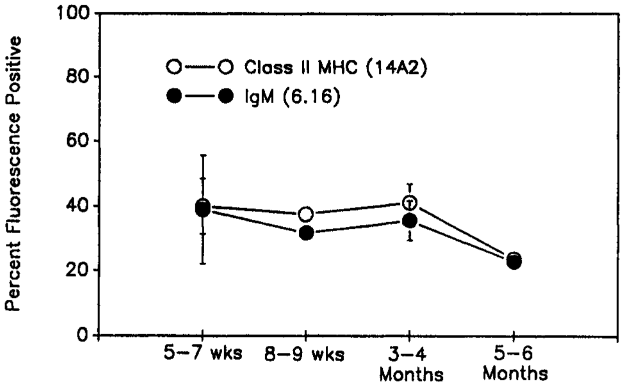

FIGURE 4. Ontogeny of expression of class II MHC antigens (O———O) and surface IgM (●———●) on splenic lymphocytes of perchlorate-blocked J-strain larvae. Staining was done as described in the legend for Fig. 1. Each data point represents the mean ± SE of 2–5 individuals or pools of animals (4–8 animals total.

TABLE 2
Dual Expression of Class II MHC antigens and IgM on Splenic Lymphocytes of Perchlorate-Treated and Untreated Control J-Strain Frogs at 5 Months of Age[a]

| Treatment | Percent class II (indirect) | Percent IgM$^+$ (indirect) | Percent class II$^+$ IgM$^+$ (direct) | Percent class II$^+$ IgM$^-$ (direct) |
|---|---|---|---|---|
| Perchlorate | 27.0 ± 5.0 | 18.3 ± 2.7 | 20.1 ± 2.0 | 2.7 ± 0.6 |
| Perchlorate | 26.8 ± 3.5 | 28.4 ± 3.4 | 28.6 ± 3.7 | 2.3 ± 1.3 |
| Untreated | 50.1 ± 3.5 | 26.6 ± 2.4 | 20.9 ± 1.5 | 12.4 ± 2.0 |
| Untreated | 63.4 ± 4.3 | 48.0 ± 0.5 | 28.1 ± 2.3 | 20.2 ± 4.4 |

[a]Data are expressed as mean ± SE of three independent counts.

### Comparison of Cell Numbers in Thymus and Spleen of Metamorphosis-inhibited Larvae and Normally Developing Controls

When preparing cells for staining, we recorded the total number of leukocytes recovered from spleen and bilateral thymuses of normal and perchlorate-treated frogs of various ages. Included in these studies were several groups of outbred larvae that developed normally or were perchlorate-treated in the same way as the J-strain animals were treated. Total leukocyte numbers in both organs in perchlorate-treated animals were not different from normal controls during larval and metamorphic stages (6–11 weeks of age). After metamorphosis, however, the number of cells in thymus and spleen of normal control frogs increased very dramatically while those in perchlorate-treated frogs remained the same or only slightly higher than those of younger larvae. At 5–6 months of age, control thymuses held six-fold more cells while control spleens held three-fold more cells (Fig. 5).

### DISCUSSION

These experiments were designed to demonstrate whether the emergence of class II$^+$ T lymphocytes in postmetamorphic frogs (Du Pasquier and Flajnik, 1990; Flajnik et al., 1990) is a metamorphosis-dependent phenomenon. Clearly, in larvae whose metamorphosis is inhibited, this population of lymphocytes does not expand. The expression of class II antigens on 8–11% of thymocytes in 3–6-month-old perchlorate-blocked larvae may suggest that in these older larvae some immature class II$^+$ thymocytes develop, but the thymic environment is not appropriate for their selection, expansion, and export. Alternatively, we cannot presently exclude the possibility that the positive cells are epithelial cells. Because most class II$^+$ cells in the spleen of these blocked animals are IgM$^+$, it is clear that very few class II$^+$ T cells reach the spleen.

The appearance of class II antigens on Ig$^-$ lymphocytes could be due to a change in the pattern of expression of class II genes on existing cells after metamorphosis or due to the appearance of a new set of cells due to lymphopoiesis after metamorphosis. The observation of Turpen and Smith (1989) that the lymphocytes of a diploid thymus implanted in a triploid host before metamorphosis were largely replaced by host cells after metamorphosis suggests a new wave of stem-cell immigration and expansion in the thymus at metamorphosis. Our comparison of the total recoverable leukocytes in untreated and perchlorate-treated frogs (Fig. 5) suggests that prevention of metamorphosis inhibits expansion of the thymic lymphocyte population that normally occurs in postmetamorphic frogs (Du Pasquier and Weiss, 1973; Rollins-Smith et al., 1984). This observation is consistent with the hypothesis that emergence of the class II$^+$ T-cell population occurs as a result of the generation of a new set of adult-type lymphocytes because of a maturational change in the thymus at metamorphosis. There are a number of structural changes observed in the thymus of *Xenopus* during metamorphosis, and failure of animals to metamorphose results in retention of larval histological features seen at the light- and electron-microscopic levels (Clothier and Balls, 1985).

The time frame in which the new adult-type T cells emerge appears to be quite broad. Although metamorphosis is generally complete by 10–12 weeks of age in our laboratory, a high proportion of class II$^+$ T cells is not observed until 5–6 months of age. Thus, it would appear that there is a period of at least two months after metamorphosis in which larval lymphocytes persist while adult-type lymphocytes are maturing. Because thymectomy just prior to metamorphosis does not significantly impair the ability of 2-month postmetamorphic frogs to reject skin allografts (Barlow and Cohen, 1983), persisting larval cells must be functional. This pattern of development may ensure that some functional tadpole cells remain to protect against pathogens, as suggested by Flajnik and Du Pasquier (1988). It raises, however, the question of how these functional tadpole cells tolerate the array of new adult-specific antigens without reacting against self. There may be suppressive mechanisms that prevent an antiself response while allowing a protective response to

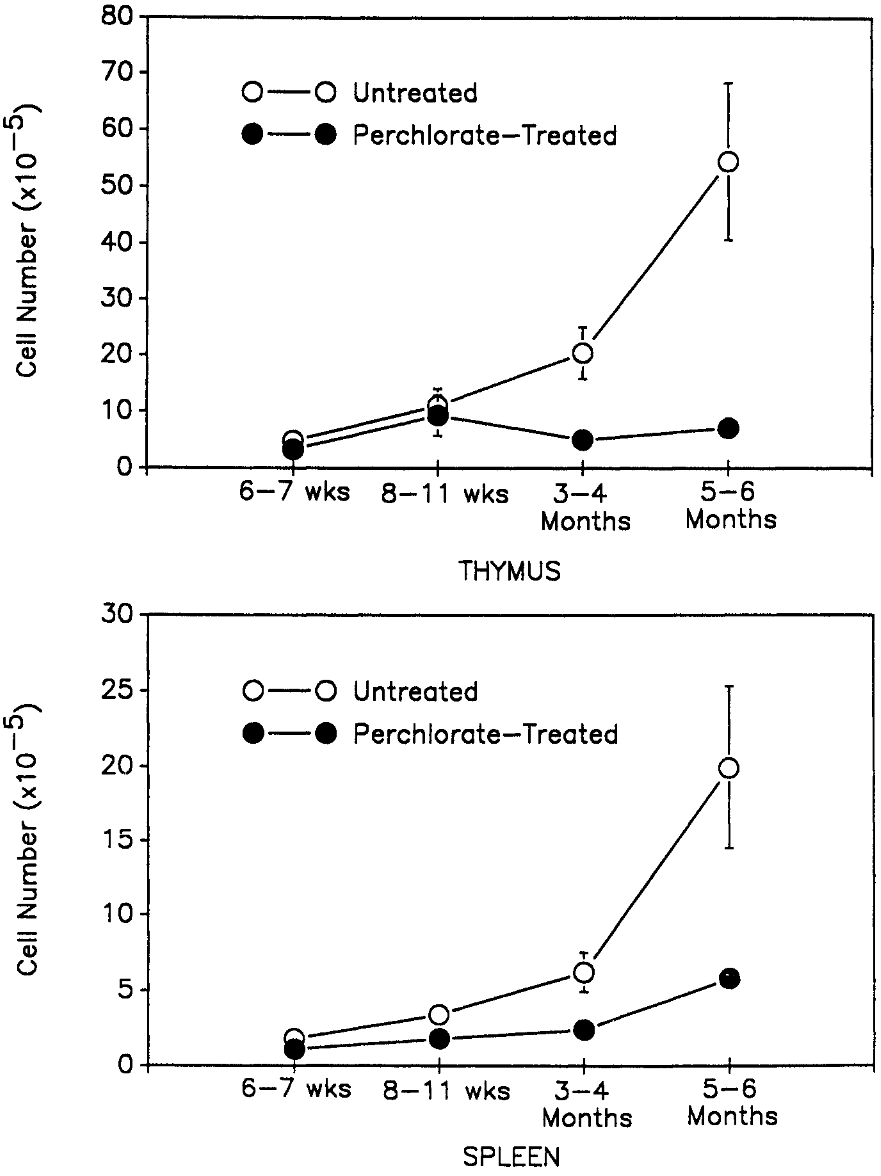

FIGURE 5. Changes in the numbers of recoverable cells in the thymus (top panel) and spleen (bottom) of untreated control (O——O) and perchlorate-treated (●——●) frogs during ontogeny. Untreated controls and perchlorate-treated animals were raised under identical conditions of animal density, temperature, nutrition, etc.. Each data point represents the mean ± SE of 5–12 individuals or pools of animals.

pathogens to occur. The phenomenon of immunosuppression around the time of metamorphosis is well documented (reviewed in Du Pasquier et al., 1989) and some type of suppressor cell appears to be involved (Du Pasquier and Bernard, 1980; Barlow and Cohen, 1983).

Our observation that development of a class II$^+$ T-cell population is metamorphosis-dependent lends support to the general hypothesis that maturation of the immune system in *Xenopus* requires a normal metamorphosis. Other features or functions of the immune system that depend on normal metamorphosis are adult-type allograft rejection (Chardonnens and Du Pasquier, 1973; DiMarzo and Cohen, 1982a, b; Obara et al., 1983; Rollins-Smith et al., 1988), and development of an adult-type antibody repertoire (Du Pasquier and Haimovich, 1976; Du Pasquier et al., 1979; Hsu and Du Pasquier, 1984b). When metamorphosis was prevented by goitrogen-treatment, the adult-type antibody pattern did not develop (Hsu and Du Pasquier, 1984b). Some other features of adult-type immunity appear to develop independently of thyroid hormone influences. Examples of this are development of high-titer IgY antibodies to a T-dependent antigen by perchlorate-blocked permanent larvae, whereas very young larvae make IgY antibodies poorly (Hsu and Du Pasquier, 1984a, b), and expression of class I MHC antigens by perchlorate-blocked larvae (Flajnik et al., 1986; Flajnik and Du Pasquier, 1988). Because expression of class I antigens is not prevented when metamorphosis is blocked, but expression of class II antigens on adult T cells is prevented, it seems clear that expression of each set of MHC antigens in adult lymphocytes is independently regulated. It seems likely, from the evidence so far accumulated, that the normal development of adult-type immunity in frogs requires a postmetamorphic expansion of both B and T lymphocytes. This expansion results in a different allograft-rejection capability and a different antibody repertoire. If metamorphosis is inhibited, the lymphocyte expansion is greatly retarded and the resulting response capability reaches a more mature larval state that does not equal the adult. Clearly, metamorphosis presents a variety of problems for the developing immune system. We are beginning to understand how the frog solves these problems, but a greater understanding awaits further study.

### MATERIALS AND METHODS

### Frogs

MHC homozygous J-strain (Tochinai and Katagiri, 1975; DiMarzo and Cohen, 1982b) males and females were induced to breed by injection of human chorionic gonadotropin according to standard procedures. Larvae were reared at approximately 8–10 tadpoles per 4 liters of dechlorinated tap water. Water was changed and the animals were fed powdered nettle leaf three times weekly. Postmetamorphic frogs were fed freeze-dried tubifex worms (Wardley Product Company, Inc., Secaucus, NJ) and small pieces of commercial trout chow (Purina, Inc., St. Louis, MO). Larval stages were determined according to the Normal Table of Nieuwkoop and Faber (1967).

### Inhibition of Metamorphosis

Beginning at 21–27 days postfertilization, some larvae were reared in dechlorinated tap water containing 1 g/l of sodium perchlorate (Sigma Chemical Co., St. Louis, MO). Water was changed and the animals were fed three times weekly.

### Monoclonal Antibodies

The monoclonal antibodies, 6.16, specific for the $\mu$ chain of *Xenopus* IgM (Bleicher and Cohen, 1981), and 14A2, specific for a polymorphic determinant on the *Xenopus* class II molecule (Flajnik et al., in press), were used for staining surface determinants on freshly prepared thymic and splenic lymphocytes. Ammonium sulfate precipitated 6.16 was used at approximately 100 $\mu$g/ml in amphibian phosphate buffered saline (APBS) (6.6 g NaCl, 1.15 g $Na_2HPO_4$, and 0.2 g $KH_2PO_4$ in 1000 ml of glass distilled water, pH adjusted to 7.4) containing 2% fetal calf serum and 0.2% sodium azide (PFA). Ammonium sulfate precipitated 14A2 or culture supernatants from the hybridoma cell line that produces it were used at approximately 200 $\mu$g/ml in PFA.

### Indirect Immunofluorescence

Approximately $5 \times 10^5$ thymocytes or splenocytes were stained with 20 $\mu$l of one of the monoclonal antibodies for 20 min at 4°C. After a wash with 0.5 ml of cold (4°C) PFA, the cells were stained with fluoresceinated goat antimouse immunoglobulin (polyvalent) antisera (Southern Biotechnology Associates, Inc., Birmingham, AL) at 100 $\mu$g/ml in PFA for an additional 20 min at 4°C. After the second antibody was washed out, the cells were resuspended at $1 \times 10^6$/ml in PFA and $1 \times 10^5$ were centrifuged onto microscopic slides with a cytocentrifuge (Shandon Southern Instrument, Inc., Sewickley, PA). After air drying, the adherent cells were fixed for 20 min in a very cold (−16°C) 95% ethanol, 5% glacial acetic acid solution. After rehydration in APBS and mounting, the slides were examined at 500–800× magnification with a Leitz Orthoplan fluorescent microscope with epillumination. The percentage of cells with positive immunofluorescence was determined after counting approximately 100–500 cells of lymphocyte morphology.

### Flow Cytometry

Cells were prepared and stained as described before and fixed for at least 1 h in PFA containing 1% formalin. Cells were analyzed on an EPICS 753 flow cytometer (Coulter Electronics, Hialeah, FL) using the 488-nm line of an argon laser operating at 25 A with 500-mW output. Fluorescent signals passed through a 457–502-nm laser-blocking filter, 515-nm absorbance filter, and a 525-nm bandpass filter prior to amplification by an RCA 4526 photomultiplier tube (PMT). Viability was determined by propidium iodide dye exclusion of an unstained cell suspension. Electronic gates were defined on forward and 90° light-scatter parameters so as to select 5000 viable cells for subsequent immunofluorescence analyses.

### Dual Fluorescence

The monoclonal antibodies, 6.16 and 14A2, were directly conjugated with fluorescein and rhodamine, respectively, according to standard protocols (Mishell and Shiigi, 1980). Each antibody stained with very dim but detectable fluorescence. To enhance the staining for microscopic examination, cells were indirectly stained with rhodamine-conjugated goat antimouse kappa light-chain-specific antibody (to detect the 14A2 monoclonal) and fluorescein-conjugated goat antimouse lambda-chain-specific antibody (to detect the 6.16 monoclonal). By this method, we were able to detect cells positive for both IgM and class II antigens.

### ACKNOWLEDGMENTS

We wish to acknowledge Dr. Wayne Green and the flow cytometry laboratory of the Veterans Administration Medical Center of Nashville for assistance with flow cytometry. We wish to thank Melanie Rardin for assistance in preparation of this manuscript.

This investigation was supported by NSF grant DCB 8710235.

*(Received February 20, 1990)*

*(Accepted April 9, 1990)*

### REFERENCES

Barlow E.H., and Cohen N. (1983). The thymus dependency of transplantation allotolerance in the metamorphosing frog, *Xenopus laevis*. Transplantation **35:** 612–619.

Bleicher P.A., and Cohen N. (1981). Monoclonal anti-IgM can separate T cell from B cell proliferative responses in the frog *Xenopus laevis*. J. Immunol. **127:** 1549–1555.

Broyles R.H. (1981). Changes in the blood during amphibian metamorphosis. *In:* Metamorphosis, a Problem in Developmental Biology, Gilbert L.I., and Frieden E., Eds. (New York: Plenum Press), pp. 461–490.

Chardonnens X., and Du Pasquier L. (1973). Induction of skin allograft tolerance during metamorphosis of the toad *Xenopus laevis:* A possible model for studying generation of self tolerance to histocompatibility antigens. Eur. J. Immunol. **3:** 569–573.

Clothier R.H., and Balls M. (1985). Structural changes in the thymus glands of *Xenopus laevis* during development. *In:* Metamorphosis, Balls M., and Bownes M.E., Eds. (Oxford: Oxford University Press), pp. 332–359.

DiMarzo S., and Cohen N. (1982a). An *in vivo* study of the ontogeny of alloreactivity in the frog, *Xenopus laevis*. Immunology **45:** 39–48.

DiMarzo S., and Cohen N. (1982b). Immunogenetic aspects of *in vivo* allotolerance induction during ontogeny of *Xenopus laevis*. Immunogenetics **16:** 103–116.

Dorn A.R., and Broyles R.H. (1982). Erythrocyte differentiation during the metamorphoic hemoglobin switch of *Rana catesbeiana*. Proc. Natl. Acad. Sci. USA **79:** 5592–5596.

Du Pasquier L., and Bernard C.C.A. (1980). Active suppression of allogeneic histocompatibility reactions during metamorphosis of the clawed toad *Xenopus*. Differentiation **16:** 1–7.

Du Pasquier L., Blomberg B., and Bernard C.C.A. (1979). Ontogeny of immunity in amphibians: Changes in antibody repertoires and appearance of adult major histocompatibility antigens in *Xenopus*. Eur. J. Immunol. **9:** 900–906.

Du Pasquier L., and Flajnik M.F. (1990). Expression of MHC class II antigens during *Xenopus* development. Dev. Immunol. 1:85–95.

Du Pasquier L., and Haimovich J. (1976). The antibody response during amphibian ontogeny. Immunogenetics **3:** 381–391.

Du Pasquier L., Schwager J., and Flajnik M.F. (1989). The immune system of *Xenopus*. Annu. Rev. Immunol. **7:** 251–275.

Du Pasquier L., and Weiss N. (1973). The thymus during the ontogeny of the toad *Xenopus laevis:* Growth, membrane-bound immunoglobulins and mixed lymphocyte reaction. Eur. J. Immunol. **3:** 773–777.

Flajnik M.F., and Du Pasquier L. (1988). MHC class I antigens as surface markers of adult erythrocytes during the metamorphosis of *Xenopus*. Dev. Biol. **128:** 198–206.

Flajnik M.F., Ferone S., Cohen N., and Du Pasquier L. (1990). *Xenopus* class II molecules. Antigenicity and unusual tissue distribution. Mol. Immunol. **27:**451–462.

Flajnik M.F., Hsu E., Kaufman J.F., and Du Pasquier L. (1987). Changes in the immune system during metamorphosis of *Xenopus*. Immunol. Today **8:** 58–64.

Flajnik M.F., Kaufman J.F., Hsu E., Manes M., Parisot R., and Du Pasquier L. (1986). Major histocompatibility complex-encoded class I molecules are absent in immunologically competent *Xenopus* before metamorphosis. J. Immunol. **137:** 3891–3899.

Hsu E., and Du Pasquier L. (1984a). Ontogeny of the immune system in *Xenopus*. I. Larval immune response. Differentiation **28:** 109–115.

Hsu E., and Du Pasquier L. (1984b) Ontogeny of the immune system in *Xenopus*. II. Antibody repertoire differences between larvae and adults. Differentiation **28:** 116–122.

Just J.J., Schwager J., and Weber R. (1977). Hemoglobin transition in relation to metamorphosis in normal and isogenic *Xenopus*. Wilhelm Roux's Arch. **183:** 307–323.

Just J.J., Schwager J., Weber R., Fey H., and Pfister H. (1980). Immunological analysis of hemoglobin transition during metamorphosis of normal and isogenic *Xenopus*. Wilhelm Roux's Arch. **188:** 75–80.

May F.E.B., and Knowland J. (1980). The role of thyroxine in the transition of vitellogenin synthesis from non-inducibility to inducibility during metamorphosis in *Xenopus laevis*. Dev. Biol. **77:** 419–430.

Mishell B.B., and Shiigi S.M., Eds. (1980). Selected Methods in Cellular Immunology Chapter 13, Section 13.3 (San Francisco: W.H. Freeman), pp. 292–297.

Nieuwkoop P.D., and Faber J. (1967). Normal Table of *Xenopus laevis* (Daudin) (Amsterdam, North Holland).

Obara N., Kawahara H., and Katagiri C. (1983). Response to skin grafts exchanged among siblings of larval and adult gynogenetic diploids in *Xenopus laevis*. Transplantation **36:** 91–95.

Rollins-Smith L.A., Parsons S.C.V., and Cohen N. (1984). During frog ontogeny, PHA and Con A responsiveness of splenocytes precedes that of thymocytes. Immunology **52:** 491–500.

Rollins-Smith L.A., Parsons S.C.V., and Cohen N. (1988). Effects of thyroxine-driven precocious metamorphosis on maturation of adult-type allograft rejection responses in early thyroidectomized frogs. Differentiation **37:** 180–185.

Tochinai, S., and Katagiri, C. (1975). Complete abrogation of immune response to skin allografts and rabbit erythrocytes in the early thymectomized *Xenopus* Dev. Growth Diff. **17:** 383–394.

Turpen J.B., and Smith P.B. (1989). Precursor immigration and thymocyte succession during larval development and metamorphosis in *Xenopus*. J. Immunol. **142:** 41–47.

